# Impact of Sarcopenia During Induction Treatment in Patients With Unresectable Locally Advanced Pancreatic Cancer

**DOI:** 10.1002/ags3.70078

**Published:** 2025-08-18

**Authors:** Sho Uemura, Masayuki Tanaka, Minoru Kitago, Hiroshi Yagi, Yuta Abe, Yasushi Hasegawa, Shutaro Hori, Yutaka Nakano, Yuko Kitagawa

**Affiliations:** ^1^ Department of Surgery Keio University School of Medicine Tokyo Japan

**Keywords:** chemotherapy, conversion surgery, sarcopenia, unresectable locally advanced pancreatic cancer

## Abstract

**Background:**

Chemotherapeutic advances have increased opportunities for conversion surgery (CS) in unresectable locally advanced (UR‐LA) pancreatic cancer (PC). However, the optimal indications for CS remain unclear. Sarcopenia has been associated with poor outcomes in PC, except UR‐LA PC. Herein, we aimed to evaluate the impact of sarcopenia on the prognosis of patients with UR‐LA PC.

**Methods:**

In this retrospective study, we reviewed consecutive patients with UR‐LA PC who had received chemo(radio)therapy as an initial treatment between 2015 and 2023. We examined relevant clinical variables and CT findings at initial diagnosis and at 6 months after starting treatment.

**Results:**

Ten of the 41 patients had undergone CS. Tumor size at 6 months, clinical lymph node metastasis at diagnosis, and changes in sarcopenia over 6 months were associated with overall survival (OS) (multivariate analysis: hazard ratio = 3.25, 2.79, and 3.51, respectively). In the entire cohort, patients without any of these three factors had significantly better OS than those with one or more (median OS: 30.3 months vs. 17.3 months, *p* = 0.013). CS was associated with better OS among patients without these factors (not reached vs. 25.5 months, *p* = 0.039), but not in those with one or more.

**Conclusions:**

The impact of change in sarcopenia on prognosis was demonstrated in patients with UR‐LA PC. Although, given the limited number of cases, CS might provide a survival benefit in carefully selected patients without prognostic factors such as tumor size at 6 months, clinical lymph node metastasis at diagnosis, and the rate of change of the psoas muscle mass index (PMI).

## Graphical abstract

This study identified three preoperative prognostic factors—tumor size at 6 months, lymph node metastasis at diagnosis, and decline in psoas muscle mass (sarcopenia)—in patients with unresectable locally advanced pancreatic cancer. Conversion surgery was associated with improved survival only in patients without these risk factors, highlighting the importance of patient selection.

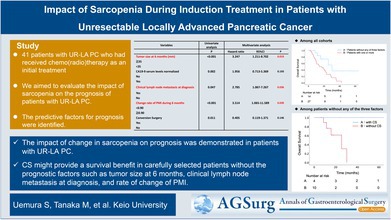

## 1 Background

Pancreatic cancer (PC) has one of the worst survival rates of cancers world‐wide. At initial presentation, only 10%–20% of patients present with resectable disease, the majority having distant metastases (50%–60%) or locally advanced disease (20%–30%) [1]. Multidisciplinary treatments combining surgical resection and chemo(radio)therapy have been implemented with the aim of improving the survival of patients with unresectable PC [2, 3].

Development of multidisciplinary treatments has enabled the performance of conversion surgery (CS) for highly selected patients with UR‐LA PC [4, 5, 6]. Several articles have reported the impacts of resectability and curative resection on the prognosis of patients who undergo CS after induction therapy [7, 8]. Although CS reportedly improves survival, the optimal indications for this procedure remain unclear [9]. Hence, in clinical practice, decisions for CS of UR‐LA PC after induction treatment have been made on a highly individualized basis.

It has recently been demonstrated that sarcopenia, which is the loss of muscle mass with age, is associated with poor short‐ and long‐term outcomes in various types of malignancies, including PC [10, 11]. An association between sarcopenia and unfavorable prognosis in patients with unresectable PC has been reported [12]. However, that series lacked evidence regarding the impact of change of sarcopenia status during chemotherapy on clinical outcomes.

Therefore, the aim of this study was to evaluate the impact of sarcopenia in the prognosis of patients with induction chemotherapy for UR‐LA PC.

## 2 Methods

### 2.1 Study Design and Participants

We retrospectively reviewed data of 41 consecutive patients with UR‐LA PC cases who received chemo(radio)therapy between January 2015 and December 2023 at the Department of Surgery, Keio University Hospital. We allocated the patients to two groups: those who had undergone pancreatectomy (CS group) and those who had not (C[R]Tx group). UR‐LA PC was defined according to the NCCN guidelines [13]. This study was approved by the Ethics Committee of the Keio University School of Medicine (Approval number: 20140389).

The most commonly administered regimens of chemo(radio)therapy were gemcitabine plus nab‐paclitaxel (GnP) or folinic acid, fluorouracil, irinotecan, and oxaliplatin (FFX), with or without radiotherapy. Decisions on this therapy were based on patient age, general condition, doctor preference, and clinical study. In the present study, we evaluated relevant clinical variables at initial diagnosis and at 6 months after commencing treatment. At least 6 months of induction treatment was generally planned for patients with newly diagnosed UR‐LA PC. For patients undergoing CS before completing 6 months of induction treatment, or discontinuing anti‐cancer therapy and transitioning to best supportive care (BSC) within 6 months, the clinical variables were evaluated at the last available time point immediately before surgery or discontinuation.

### 2.2 Patient Surveillance During Chemo(Radio)therapy

From the time of initial diagnosis and commencement of chemo(radio)therapy, patients were generally reevaluated every 3 months during treatment by clinical examination, measurement of CA19‐9 serum levels, and computed tomography (CT).

The imaging data were transferred to a computer workstation to assess the psoas muscle area. Cross‐sectional areas of the right and left psoas muscles at the middle level of the third lumbar vertebra were automatically measured using SYNAPSE VINCENT software (Fuji Film, Tokyo, Japan). The measured psoas muscle area was normalized for height squared (m^2^) using the following equation: normalized psoas muscle area, defined as the psoas muscle mass index (PMI, cm^2^/m^2^) = measured psoas muscle area (cm^2^) / height^2^ (m^2^) [14].

### 2.3 Indications for Conversion Surgery

The surgical indications for UR‐LA PC patients were discussed by a multidisciplinary team of surgeons, oncological physicians, and radiologists. The general surgical indications were: (1) no tumor progression on imaging; (2) declining or stable CA19‐9 levels; and (3) technically resectable disease (including the consideration of venous and arterial resections). The final decisions concerning performing CS on patients who did not fulfil all of these criteria were made by the multidisciplinary team.

### 2.4 Outcomes

Study variables included patient characteristics and laboratory data. Normal CA19‐9 serum levels were defined as ≤ 37 U/mL at the relevant assessment point. Tumor size was defined as the maximum tumor diameter on axial CT slices. Clinical lymph node metastasis was defined as nodes having a short axis of 10 mm or more on CT images or showing increased uptake on PET‐CT. Inflammation‐based prognostic scores, which indicate nutritional status and predict prognosis, such as the Modified Glasgow Prognostic Score (mGPS), controlling nutritional status (CONUT), the prognostic nutritional index (PNI), neutrophil–lymphocyte ratio (NLR), and platelet–lymphocyte ratio (PLR), were calculated [15, 16, 17, 18, 19]. A rate of change of PMI was calculated by dividing the PMI at 6 months after initial treatment by the PMI at diagnosis (PMI at 6 months after initial treatment/PMI at diagnosis). A value less than 1.0 indicates a reduction in muscle mass during treatment, reflecting progression of sarcopenia. All continuous variables were divided by the appropriate cutoff value to convert them to categorical variables.

The primary endpoint of this study was determination of predictive factors of overall survival (OS) in patients with UR‐LA PC.

### 2.5 Statistical Analysis

All analyses were performed using SPSS software (version 29; IBM Corp., Armonk, NY, USA). In the C[R]Tx group, progression‐free survival (PFS) was estimated from the start of induction treatment until radiologic progression or death. OS was defined as the time from the date of initiation of chemo(radio)therapy to the date of death from any cause and was calculated using Kaplan–Meier survival analysis. Data were censored if the patient was alive at the time of the analysis or had been lost to follow‐up. The optimal cutoff points for continuous variables for OS were determined by using a minimum *p* value approach [20]. All possible cutoff points were examined by means of the log‐rank test. The value with the minimum *p* value was considered to be the optimal cutoff point. We performed univariate analyses using Cox proportional hazard regression models to select variables likely related to OS among all candidate factors. Factors with *p* < 0.05 according to univariate analysis were used in the multivariate analysis with Cox proportional hazard regression models to identify independent prognostic factors.

## 3 Results

### 3.1 Baseline Patient Characteristics and Survival Outcomes

**FIGURE 1:**
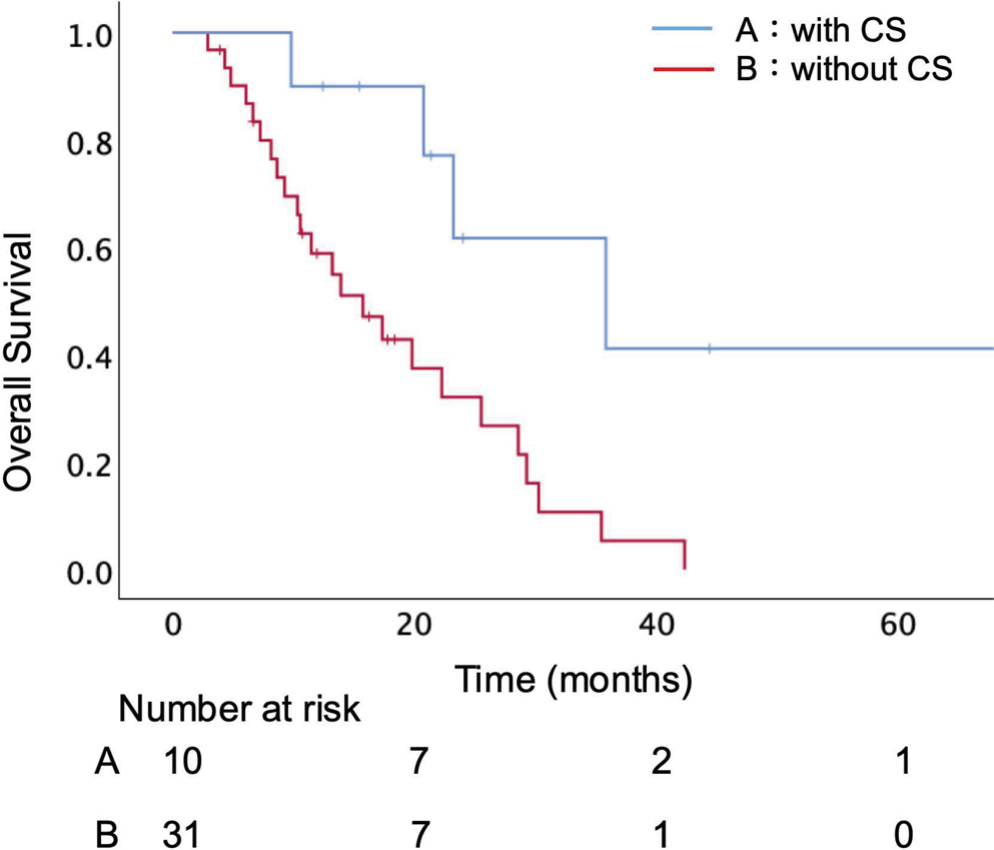
Overall survival of all patients who had undergone CS vs. had not undergone CS. There was a significant difference (*p* = 0.006). CS, Conversion surgery.

Forty‐one patients with UR‐LA PC who received chemo(radio)therapy as the initial treatment were identified (Table 1). Preoperative CA19‐9 serum levels were not available for two of these patients (5%). The median data of the following variables tended to worsen at 6 months after starting treatment: PMI, mGPS, CONUT, PNI, NLR, and PLR. The distribution of the PMI change rate during the first 6 months after treatment, stratified by CS status, is shown in Figure S1. Surgery was scheduled for 14 patients (34%) and CS on 10 (24%). Four patients underwent palliative or exploratory surgery, due to two cases of peritoneal dissemination, one cytology‐positive peritoneal washings, and one increased local invasion that was found intraoperatively. Details of the 10 patients who underwent CS are summarized in Table 2. Among these, seven patients required vascular resection, and R0 resection was achieved in five patients. Two patients received induction therapy for more than 6 months. There were no patients who changed regimens due to an unfavorable response to the initial chemotherapy. However, four patients received GnP followed by S‐1 and radiotherapy based on our clinical trial protocol (UMIN ID: 000034410), although they were not enrolled in the trial due to eligibility criteria. Comparisons between the CS and the C[R]Tx groups revealed that no significant differences in terms of CA19‐9 at diagnosis and at 6 months. The CS group had significantly smaller tumor sizes at 6 months (15 mm vs. 33 mm, *p* = 0.030) and greater PMI at 6 months (5.46 cm^2^/m^2^ vs. 4.76 cm^2^/m^2^, *p* = 0.025). The median OS was longer in the CS than C[R]Tx group (35.9 months vs. 15.7 months, *p* = 0.006) (Figure 1). In the C[R]Tx group, the median PFS was 10.4 months (range: 0.6–56.8). Nineteen patients (61%) maintained progression‐free status for at least 6 months.

**TABLE 1:** Patient background of all patients and according to resection status.

Parameter	Patients *N* = 41	CS group *N* = 10	C[R]Tx group *N* = 31	*p*
Age (years), median (range)	69 (42–86)	72 (57–77)	69 (42–86)	0.988
Male gender, *n* (%)	24 (58.5)	8 (80.0)	16 (51.6)	0.096
Body mass index (kg/㎡), median (range)	21.7 (16.8–30.6)	22.2 (19.1–29.3)	20.6 (16.8–30.6)	0.115
Chemotherapy regimen				
Gemcitabine base, *n* (%)	35 (85.4)	9 (90.0)	26 (83.9)	0.644
5‐FU base, *n* (%)	5 (12.2)	1 (10.0)	4 (12.9)	0.813
RTx, *n* (%)	17 (41.5)	4 (40.0)	13 (41.9)	0.917
Clinical lymph node metastasis at diagnosis, *n* (%)	11 (26.8)	2 (20.0)	9 (29.0)	0.586
CA19‐9 (U/ml)				
At diagnosis, median (range)	104 (2–5831)	49 (12–742)	115 (2–5831)	0.492
After 6 months, median (range)	48 (2–20 548)	19 (7–90)	76 (2–20 548)	0.402
Tumor size (mm)				
At diagnosis, median (range)	34 (14–100)	33 (15–45)	34 (14–100)	0.629
After 6 months, median (range)	30 (7–100)	15 (11–37)	33 (7–100)	0.030
PMI (cm^2^/m^2^)				
At diagnosis, median (range)	5.27 (3.37–8.06)	6.07 (4.01–8.06)	5.18 (3.37–7.90)	0.120
After 6 months, median (range)	4.95 (2.13–7.68)	5.46 (3.84–7.68)	4.76 (2.13–7.27)	0.025
Rate of change in PMI during the first 6 months, median (range)	0.88 (0.38–1.06)	0.93 (0.79–0.99)	0.83 (0.38–1.06)	0.014
mGPS (0/1/2)				
At diagnosis	33/7/1	8/2/0	25/5/1	0.883
After 6 months	19/12/10	9/1/0	10/11/10	0.112
CONUT (normal/mild/moderate/severe)				
At diagnosis	14/22/5/0	5/2/3/0	9/20/2/0	0.969
After 6 months	5/12/18/6	4/4/2/0	1/8/16/6	0.106
PNI				
At diagnosis, median (range)	47.2 (37.1–55.8)	50.9 (41.2–52.8)	45.6 (37.1–55.8)	0.249
After 6 months, median (range)	42.2 (29.2–52.0)	46.3 (38.9–50.7)	39.3 (29.2–52.0)	0.229
NLR				
At diagnosis, median (range)	2.8 (1.1–7.5)	2.7 (1.4–5.2)	2.9 (1.1–7.5)	0.651
After 6 months, median (range)	3.7 (0.7–17.3)	3.7 (1.2–7.5)	3.6 (0.7–17.3)	0.056
PLR				
At diagnosis, median (range)	172.5 (91.0–387.0)	127.4 (93.9–288.1)	179.6 (91.0–387.0)	0.235
After 6 months, median (range)	241.3 (46.4–1168.0)	232.8 (99.2–344.7)	244.5 (46.4–1168.0)	0.234

Abbreviations: 5‐FU, 5‐fluorouracil; CA19‐9, carbohydrate antigen 19–9; CONUT, controlling nutritional status; C[R]Tx, chemo(radio)therapy; CS, conversion surgery; mGPS, modified Glasgow Prognostic Score; NLR, neutrophil–lymphocyte ratio; PLR, platelet–lymphocyte ratio; PMI, psoas muscle mass index; PNI, prognostic nutritional index; RTx, radiation therapy.

**TABLE 2:** Details of Patients who underwent Conversion Surgery.

No.	Age/Gender	Tumor location	Duration of CR[T]x (months)	Regimen(s)	Operation	Vascular resection	Curability	Therapeutic effect	cN	ycN	pN
1	72 / M	Ph	3	GnP → S‐1 + RTx	SSPPD	−	R0	Grade II	−	−	−
2	77 / M	Pb	2	GnP	DP‐CAR	CA	R1	Grade Ib	−	−	+
3	57 / M	Ph	5	GnP → S‐1 + RTx	SSPPD	−	R1	Grade II	+	−	+
4	53 / M	Ph	4	GnP → S‐1 + RTx	SSPPD	SMV	R0	Grade III	−	−	−
5	75 / M	Ph	11	GnP → S‐1 + RTx	SSPPD	SMV	R1	Grade II	−	−	−
6	71 / M	Ph	6	GnP	SSPPD	−	R0	Grade Ib	−	−	+
7	74 / M	Pb	2	GnP + RTx	DP‐CAR	CA	R0	Grade Ib	+	+	+
8	76 / F	Pb	4	GnP + RTx	DP‐CAR	CA	R0	Grade II	−	−	−
9	66 / M	Pb	3	GnP	DP‐CAR	CA	R1	Grade Ib	+	+	+
10	57 / F	Ph	5	mFOLFORINOX	SSPPD	SMV	R1	Grade II	+	−	−

Abbreviations: CA, celiac artery; cN, clinical lymph node metastasis; CR[T]x, chemo(radio)therapy; DP‐CAR, distal pancreatectomy with celiac axis resection; Pb, pancreatic body; Ph, pancreatic head; pN, pathological lymph node metastasis; R0, curative resection with negative margin; R1, microscopic residual tumor; RTx, radiotherapy; SMV, superior mesenteric vein; SSPPD, subtotal stomach‐preserving pancreaticoduodenectomy; ycN, post‐therapy clinical lymph node metastasis.

### 3.2 Defining Cutoff Points

According to a minimum *p* value approach using the log‐rank test, in which every possible cutoff point for each continuous variable for OS was assessed, continuous variables were converted to dichotomous variables using the relevant cutoff points. The best cutoff point for tumor size at 6 months was 35 mm (*p* < 0.001), and that for the rate of change of PMI during the first 6 months was 0.90 (*p* < 0.001).

### 3.3 Univariate and Multivariate Analysis of Survival

According to univariate analysis, tumor size at 6 months, normalization of CA19‐9 serum levels, clinical lymph node metastasis at diagnosis, rate of change in PMI during the first 6 months, and CS were significantly associated with OS (*p* < 0.001, *p* = 0.002, *p* = 0.047, *p* < 0.001, and *p* = 0.011, respectively). Among these five factors, multivariate analysis revealed that tumor size at 6 months, clinical lymph node metastasis at diagnosis, and rate of change of PMI during the first 6 months were significantly relevant to OS (*p* = 0.019, *p* = 0.036, and *p* = 0.039, respectively) (Table 3).

**TABLE 3:** Results of univariate and multivariate analysis of predictors of overall survival.

Variables	Univariate analysis	Multivariate analysis		
	*p*	Hazard ratio	95% CI	*p*
Tumor size at 6 months (mm)	< 0.001	3.247	1.211–8.702	0.019
≧ 35				
< 35				
CA19‐9 serum levels normalized	0.002	1.956	0.713–5.369	0.193
No				
Yes				
Clinical lymph node metastasis at diagnosis	0.047	2.785	1.067–7.267	0.036
Yes				
No				
Change rate of PMI during 6 months	< 0.001	3.514	1.065–11.589	0.039
< 0.90				
≧ 0.90				
Conversion Surgery	0.011	0.405	0.119–1.371	0.146
Yes				
No				

Abbreviation: PMI, psoas muscle mass index.

### 3.4 Prognostic Stratification Based on the Presence of Risk Factors and the Effect of Conversion Surgery

**FIGURE 2:**
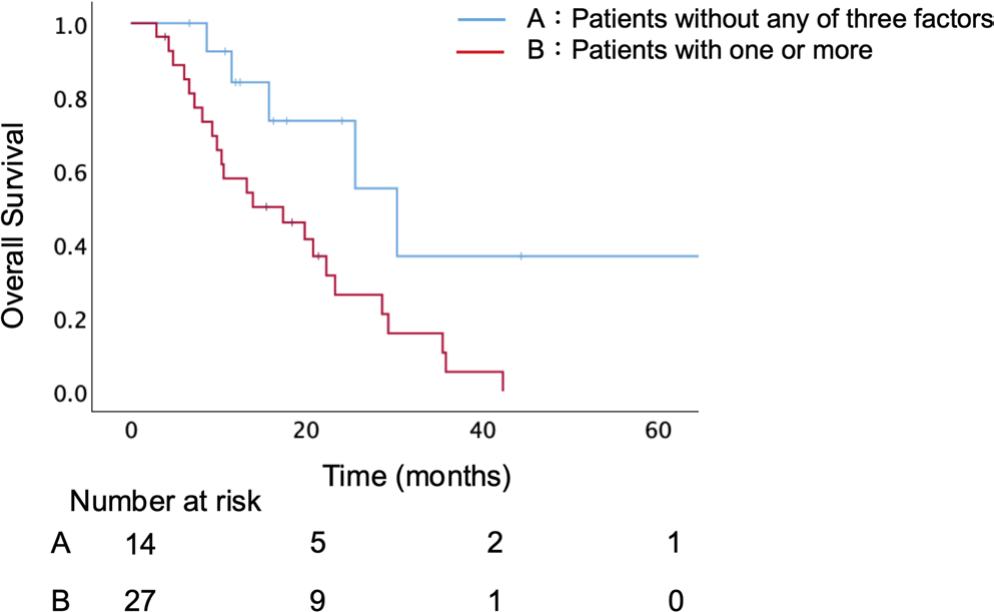
Overall survival stratified by the presence or absence of three independent poor prognostic factors. Patients without any of these factors (A) had significantly longer OS than those with one or more (B) (30.3 months vs. 17.3 months, *p* = 0.013). PMI, psoas muscle mass index.

**FIGURE 3:**
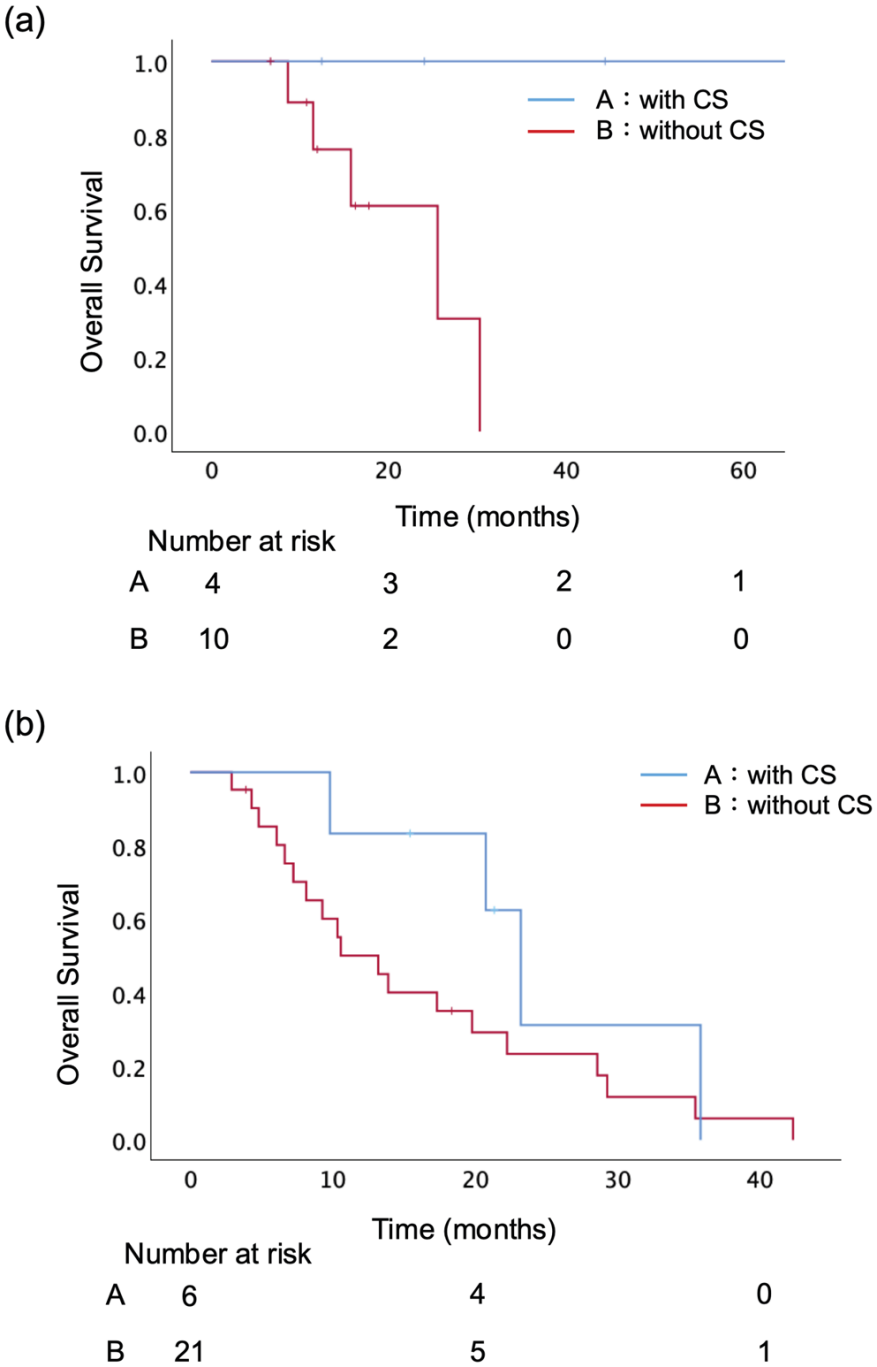
Effect of conversion surgery (CS) stratified by the presence of poor prognostic factors. (a) Among patients without any of the three factors, those who underwent CS had significantly longer OS than those who did not (not reached vs. 25.5 months, *p* = 0.039). (b) Among patients with one or more of the three factors, CS was not associated with a significant survival benefit (23.2 months vs. 13.2 months, *p* = 0.183). CS, conversion surgery.

According to these three factors identified by the multivariate analysis, 14 patients (34%) had none of these factors, while 27 patients (66%) had at least one factor. In 41 UR‐LA PC patients, there was a significant difference in OS between those without any of these factors and those with one or more (median OS: 30.3 months vs. 17.3 months, *p* = 0.013) (Figure 2). In the subgroup analysis, 4 underwent CS among the 14 patients without any of the three factors, and CS was significantly associated with improved survival (median OS: not reached vs. 25.5 months, *p* = 0.039; Figure 3a). On the other hand, in the patients with one or more factors, CS did not significantly improve survival (median OS: 23.2 months vs. 13.2 months, *p* = 0.183; Figure 3b).

## 4 Discussion

Rate of change of PMI during the first 6 months after initial treatment, in addition to tumor size at 6 months and clinical lymph node metastasis at diagnosis, was identified as independent predictors of survival in patients with initially UR‐LA PC who received chemo(radio)therapy. Patients without any of these three poor prognostic factors had significantly better OS compared to those with one or more in all patients.

Predictive factors of the prognosis of patients who undergo induction treatment followed by CS for UR‐LA PC have been controversial over the past decade [21]. With advances in multidisciplinary treatments for UR‐LA PC, several prognostic factors, such as normalization of CA19‐9 levels after induction treatment, tumor shrinkage, and CS, have been reported [8, 22]. Additionally, sarcopenia, as a poor prognostic factor, has been given attention to [12, 23]. Although several studies have reported indications for CS, no association between sarcopenia and CS has been reported.

In the present study, we identified these three factors as being significantly associated with prognosis. Tumor size after induction treatment as a predictor of resectability and OS has been reported in several articles [9, 23, 24]. Regional lymph node metastasis has been shown to be associated with poor prognosis in patients with resectable, borderline resectable, and UR‐LA PC [25, 26, 27]. Progression of sarcopenia has been reported to impair patients' tolerance to chemotherapy [28, 29], so that its dose reductions or discontinuation are often needed. Deterioration in skeletal muscle mass during neoadjuvant therapy has been linked to poor prognoses in patients with resectable and borderline resectable PC, but has not been reported in those with UR‐LA PC [28, 30]. This is the first study to identify sarcopenia during induction chemotherapy as a prognostic factor in patients with UR‐LA PC. Furthermore, the present finding suggests that evaluation of changes in muscle mass may also guide indications for CS.

While previous studies have suggested various clinical criteria for selecting candidates for CS [8, 9, 21, 22, 31], CS itself was not identified as an independent prognostic factor in the overall cohort. This result may reflect our suboptimal selection of surgical candidates. In addition, the relatively small number of patients and low R0 resection rate (50%) may have limited the statistical power to detect a significant prognostic impact of CS. Nevertheless, the subgroup analysis revealed that CS was associated with improved survival in patients without any of the three poor prognostic factors. These findings indicate that CS may provide a survival benefit when offered to carefully selected patients, although further validation is required.

This study had several limitations. First, it was a small, retrospective, single‐institution study. As with previous studies, the limited number of CS cases makes it difficult to establish a validation cohort [9, 30]. To our knowledge, no prior studies have incorporated a validation cohort for CS indications due to the small sample size of CS cases. The novelty of our study lies in identifying sarcopenia as a prognostic marker in patients with UR‐LA PC. However, large, multi‐institutional cohorts are needed for validation. Second, although the duration of this study was approximately 6 months based on the previous reports, the optimal duration of induction treatment for CS varied widely [31, 32, 33]. Third, clinical parameters were basically evaluated at 6 months after commencing treatment. However, the variations in timing of evaluation of clinical parameters in patients who underwent CS or selected BSC within 6 months may have contributed to a risk of bias. Eight patients in this cohort discontinued anticancer therapy and transitioned to BSC within 6 months. For these patients, clinical parameters were evaluated at the last available time point before the transition. Their worsening clinical status during the BSC period might have influenced the deterioration of nutritional and inflammatory indices, potentially introducing bias in the prognostic evaluation. Fourth, induction treatment regimens were not standardized. This may lead to the heterogeneity of morphologic response by various agents. Finally, comparison between the surgical and nonsurgical groups is inherently biased, as resected patients were those who responded to therapy and became technically resectable, while nonresected patients did not. Importantly, the indication for CS was determined not only by imaging but also by intraoperative findings, and there were no patients deemed resectable intraoperatively who did not proceed to surgery.

## 5 Conclusions

In this study, the impact of sarcopenia during induction chemotherapy on prognosis was clarified for the first time in UR‐LA PC. Although the number of cases was limited, CS may offer a survival benefit in carefully selected patients without prognostic factors such as tumor size at 6 months, lymph node metastasis at diagnosis, and sarcopenia progression.

## Author Contributions

**Sho Uemura:** writing – original draft, investigation. **Masayuki Tanaka:** writing – review and editing, writing – original draft. **Minoru Kitago:** writing – review and editing, writing – original draft. **Hiroshi Yagi:** data curation, writing – review and editing. **Yuta Abe:** data curation, writing – review and editing. **Yasushi Hasegawa:** writing – review and editing, data curation. **Shutaro Hori:** writing – review and editing, data curation. **Yutaka Nakano:** writing – review and editing, data curation. **Yuko Kitagawa:** writing – review and editing, data curation.

## Ethics Statement

This study was approved by the Ethics Committee of the Keio University School of Medicine (Approval number: 20140389).

## Conflicts of Interest

None of the authors has any conflicts of interest to declare. Y. Kitagawa is the editor‐in‐chief of the Annals of Gastroenterological Surgery. He was not involved in the decision to accept this article for publication.

## Supporting information


**Figure S1.** Distribution of the rate of change in psoas muscle mass index during the first 6 months after initial treatment in patients who underwent conversion surgery (CS group) and those who did not (C[R]Tx group). A box plot illustrates that the CS group showed a trend toward better PMI preservation (*p* = 0.014). CS, conversion surgery; C[R]Tx, chemo(radio)therapy.
