# Supplementary figures and images for: Impact of Sarcopenia During Induction Treatment in Patients With Unresectable Locally Advanced Pancreatic Cancer
Source: Ann Gastroenterol Surg. 2025 Aug 18;10(1):211–8. doi: 10.1002/ags3.70078 (PMC12757159; doi:10.1002/ags3.70078)

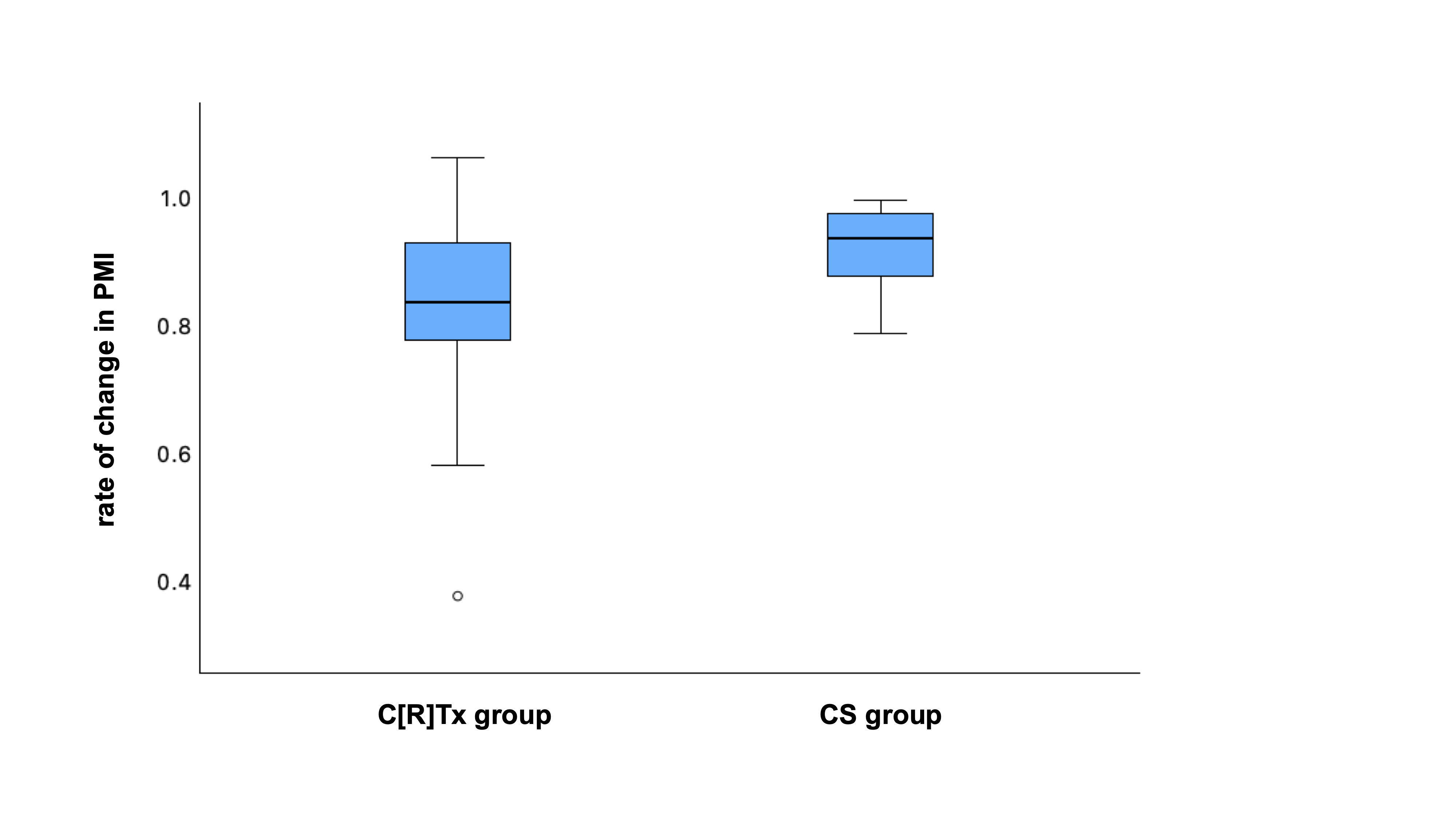

Supplement: Supplementary file 1 — Figure S1. Distribution of the rate of change in psoas muscle mass index during the first 6 months after initial treatment in patients who underwent conversion surgery (CS group) and those who did not (C[R]Tx group). A box plot illustrates that the CS group showed a trend toward better PMI preservation (p = 0.014). CS, conversion surgery; C[R]Tx, chemo(radio)therapy. [file AGS3-10-211-s001.tiff]
